# A179 MECHANISMS OF ACTION INVOLVED IN THE WOUND HEALING EFFECT OF THE DIETARY FIBRE RHAMNOGALACTURONAN

**DOI:** 10.1093/jcag/gwac036.179

**Published:** 2023-03-07

**Authors:** C Baggio, J Shang, A M Nascimento, T R Cipriani, W MacNaughton

**Affiliations:** 1 University of Calgary, Calgary, Canada; 2 Federal University of Acre, Cruzeiro do Sul; 3 Federal University of Parana, Curitiba, Brazil

## Abstract

**Background:**

Inflammatory bowel diseases (IBD) are chronic and relapsing inflammatory conditions associated with impaired intestinal epithelial barrier. Mucosal healing is the primary goal for IBD treatment since it is a good predictor of clinical remission. We previously showed the direct beneficial effects of rhamnogalacturonan (RGal), a polysaccharide isolated from the plant *Acmella oleracea*, on intestinal epithelial barrier function with participation of TLR4 and PKC activation. RNAseq data and pathway analysis have indicated the involvement of the canonical nuclear factor kB (NF-kB) signaling pathway.

**Purpose:**

We hypothesize that RGal increases intestinal epithelial wound healing through FAK-Src/PI3K/NF-kB signaling pathways.

**Method:**

Caco-2 and T84 cells, and human primary cell monolayers grown from ulcerative colitis patient-derived organoids, were wounded and treated with vehicle (media or 0.5% DMSO in media) or RGal (1000 μg/ml) for 48 h. Wound healing was assessed using either the IncuCyte or ImageXpress Pico live cell imaging systems. Proliferation and apoptosis of cells were evaluated using EdU and TUNEL assays, respectively. Inhibitors were added at the same time (transcription inhibitor Actinomycin D, 5 μg/ml) or 1 h (FAK inhibitor FAK-14, 10 μM, Src inhibitor PP2, 5 μM, PI3K inhibitor LY294002, 20 μM, NF-kB inhibitors Bay 11-7082 and JSH-23, 10 and 20 μM, respectively, or COX-2 inhibitor NS-398, 20 μM) before RGal treatment. Unwounded Caco-2 monolayers treated with RGal (1000 μg/ml) were collected for Western blotting for COX-2 protein.

**Result(s):**

In the wound healing assay, RGal (1000 μg/ml) enhanced wound healing by 12.5% at 48 h in Caco-2 cells and by 14.7% at 24 h in T84 cells, compared to control group. RGal (1000 μg/ml) also accelerated the wound closure in colonoid monolayers obtained from ulcerative colitis patient biopsies by 81.3% at 48 h. Neither proliferation nor apoptosis were involved in the RGal effect on wound healing. Treatment of cells with FAK14 (10 μM), PP2 (5 μM), and PI3K (20 μM) significantly prevented the RGal-induced wound healing. Actinomycin D (5 μg/ml), Bay 11-7082 (10 μM) or JSH-23 (20 μM) treatment significantly reversed the effect of RGal on wound healing, showing that the response was transcriptionally dependent and involved NF-kB signaling. Treatment of cells with NS-398 (20 μM) also reversed the effect of RGal on wound healing. COX-2 protein expression was significantly increased at 6 and 12 h after RGal addition to Caco-2 monolayers.

**Conclusion(s):**

These data suggest that the plant-based polysaccharide RGal increases intestinal epithelial cell wound healing by increasing cell migration. The RGal effect is dependent on the activation of the FAK-Src/PI3K signaling pathways and subsequently the transcription factor NF-kB and downstream COX-2 protein expression and activity. Our findings show a novel mechanism of action of RGal in wound healing that could help in the resolution of intestinal inflammation and mucosal healing.

**Please acknowledge all funding agencies by checking the applicable boxes below:**

Other

**Please indicate your source of funding;:**

NSERC

**Disclosure of Interest:**

None Declared

